# Socio-economic drivers of drug-resistant tuberculosis in Africa: a scoping review

**DOI:** 10.1186/s12889-021-10267-0

**Published:** 2021-03-11

**Authors:** Lesley-Ann Lynnath Cannon, Kelechi Elizabeth Oladimeji, Daniel Ter Goon

**Affiliations:** Department of Public Health, Faculty of Health Sciences, University of Fort Hare, East London, 5200 Eastern Cape, South Africa

**Keywords:** Drug-resistant TB, Socio-economic drivers, Africa

## Abstract

**Background:**

Drug-resistant TB **(**DR-TB) remains a public health concern due to the high morbidity and mortality rates from the disease. The DR-TB is a multifaceted illness with expensive treatment regimens, toxic medications and most often the long duration of treatment constitutes a substantial financial burden on both infected patients and the health system. Despite significant research advances in the diagnosis and treatment, there is a paucity of synthesized evidence on how socio-economic factors are associated with DR-TB. This review aims to address this gap by synthesizing available evidence and data on the common socio-economic drivers of DR-TB infection in Africa.

**Methods:**

A systematic search was conducted on PUBMED and Google Scholar databases from January 2011 to January 2020 using Joanna Briggs Institute’s scoping review approach. An updated search was conducted on 21 September 2020. The eligibility criteria only included systematic reviews and studies with quantitative research methods (cross-sectional, case-control, cohort, and randomized-control trials). Studies conducted in Africa and focusing on socio-economic factors influencing DR-TB burden in African countries were also considered. Data was extracted from all the studies that met the eligibility criteria based on the study’s objectives.

**Results:**

Out of the 154 articles that were retrieved for review, 20 abstracts of these articles met all the eligibility criteria. Of the 20 articles, 17 quantitative and 3 reviews. Two additional articles were found eligible, following the updated search. The following themes were identified as major findings: Social and economic drivers associated with DR-TB. Substance abuse of which, stigma and discrimination were the prominent social drivers. Economic drivers included poverty, financial constraints because of job loss, loss of productive time during hospital admission and treatment costs.

**Conclusion:**

This review has highlighted which socio-economic factors contribute to DR- TB This is relevant to assist DR-TB management program and TB stakeholders in different settings to address identified socio-economic gaps and to reduce its negative impact on the programmatic management of DR TB. Therefore, redirecting strategies with more focus on socio-economic empowerment of DR-TB patients could be one of the innovative solutions to reduce the spread and eliminate DR-TB in Africa.

**Supplementary Information:**

The online version contains supplementary material available at 10.1186/s12889-021-10267-0.

## Background

Tuberculosis (TB) is a global public health concern and one of the leading causes of morbidity and mortality worldwide [1–3]. Drug-resistant TB (DR-TB) has evolved due to a high TB defaulter rate, leading to complications with TB diagnosis and case management especially in low- and middle-income countries [4–6]. As described in the World Health Organization (WHO) global TB report for 2019, roughly 500,000 persons (range 417,000–556,000) developed or acquired rifampicin-resistant TB (RR-TB) and of these, 78% had multidrug-resistant TB (MDR-TB). Of all newly reported TB cases globally, a projected 3.4% had RR−/ MDR-TB while among previously treated patients, 18% had RR−/MDR-TB [7]. Further, there is an increase in DR-TB case notification worldwide as indicated in the total number of 186,772 cases of MDR/RR-TB detected and notified in 2018 when compared to the 160,684 notified DR TB cases in 2017. Similarly, increased number of DR-TB cases (156,071 in 2018 compared to 139,114 in 2017) commenced treatment Even with this increased number placed on treatment, treatment success globally remains low, at 56% [7].

The precise burden of DR-TB in the African countries is poorly described; with only 51% of countries having done official reviews [8]. Thus, DR-TB is commonly missed in Africa and this looms prospects to attain the year 2035 targets of the End TB strategy [9]. This can lead to difficulty in estimating the accurate degree of the problem in the African Region. Available data reveals that between 36,000 and 44,000 MDR-TB cases were reported in the AFRO Region in 2016 and of these, 15% of new MDR-TB patients were found to have RR [3]. Empirical evidence indicates that DR-TB is a multifaceted illness with expensive treatment regimens, toxic medications and long duration of treatment creating a substantial financial burden on the health system [10, 11]. Consequently, socio-economic status, job loss, overpopulation, poor hygiene, immunocompromising illnesses and malnourishment have been identified as risk factors that influence this disease burden hence it is commonly referred to as the disease of the poor [12]. Other facilitators of the disease burden influencing the disease management and outcomes include treatment inaccessibility and distance to the healthcare facility, transport expenses and costs experienced during hospitalization [9, 13–15]. Though significant research developments have been made in the diagnosis and treatment aspects of DR-TB [16], research focused on socio-economic factors associated with DR-TB are limited.

### Scoping review research question

Drug resistant TB is a huge public health problem as discussed, hence this review set out to answer these questions:

1. What are the social factors that contribute to the burden of DR- TB and treatment outcomes in Africa?
2. What are the economic factors influencing the burden of DR-TB and treatment outcomes in Africa?

### Scoping review objective

The objective of this review was to synthesize published articles to identify socio-economic drivers contributing to the burden of DR- TB in Africa.

## Methods

This scoping review was conducted using the Preferred Reporting Items for Systematic Reviews and Meta-Analyses (PRISMA) for scoping review [17] and framework by Joanna Briggs Institute (JBI) [18]. This review was not registered in the Prospective Register of Systematic Reviews (PROSPERO) since it is a scoping review and not a systematic review. However, there is the prospect of change in trend where registration for scoping review becomes mandatory in databases like PROSPERO, Open Science Framework and Figshare [18]. This scoping review was therefore registered in the Open Science Framework (https://osf.io/m23pk).

### Search strategy

PubMed and Google Scholar databases were searched to retrieve articles published between the year 2011 and 2020. Accordingly, only studies meeting the eligibility criteria discussed below was included in the review. The initial search was done on 7 January 2020 and an updated search on 21 September 2020. The following search terms and keywords were used; socio-economic factors, drug-resistance TB, multidrug-resistant tuberculosis, extremely/extensively drug-resistant TB, Africa, social factors, economic factors, contributing factors, and risk factors. During the data sources search process, sorting by year of publication was first applied and next, we only considered the first 10 pages of the search results due to multiple similarities (duplicates) and unrelated articles (not about TB/ not SES).

### Eligibility criteria

#### Inclusion criteria

Since this is a scoping review, we used the PCC (Population / Concept / Context) framework recommended by JBI [18] to identify eligible studies as discussed below.

- **Population (P)** – Drug Resistant TB patients
- **Concept (C)** – quantitative studies investigating the influence of socio- economic factors/ drivers/ status on treatment outcomes such as adherence/ cure and treatment completed rates/ Death rates/ Reinfections.
- **Context (C)** – we carried out our search process between 1 January 2011 to 7 January 2020. Only cohort, case-control, cross-section studies and systematic reviews evaluating socio-economic factors influencing adult DR-TB patients from African countries were included for further screening and synthesis. An updated search was carried out on 21 September 2020.

Exclusion criteria

1. Publication not peer-reviewed
2. Studies on drug sensitive TB and general TB were excluded
3. Other reviews (editorial, commentaries), book chapters, editorials, letters, and conference abstracts.
4. Publication of any other language than English.
5. Qualitative studies
6. Studies including children under 12 years and participants in prison.

### Selection process

We adopted a selection process described by the Joanna Briggs Institute online manual. Two reviewers independently searched for articles using the eligibility criteria. Articles retrieved were exported into MS Excel sheet by both reviewers and assessed for duplication and eligibility. There were no discrepancies in articles selected and screened for inclusion in this review.

### Data extraction and analysis

Two review authors (LLC/ KEO) independently screened titles and abstracts of the articles for eligibility using the earlier discussed criteria. Full text screening was then further carried out on identified eligible studies from title and abstract screening. Both the reviewers selected the studies based on full text review. Data was thereafter extracted based on the aim of this study and thematically analyzed. The data extracted is presented in Table 1 and addendum 3 and includes author(s)/, year of publication, country of origin (where the study was published or conducted), methodology, study population, objectives, findings, implications and main SE factors. Themes that emerged will be discussed in the findings and discussion section.

**Table 1:** Characteristics of the study population

Characteristics	N	%	Citation
Mean age	**33, 7**	**–**	[19–37]
**Sex**			
Male	**276,751**	**66,9**	[19–36, 38–40]
Female	**137,025**	**33,1**	[19–36, 38–40]
**Type of DR TB**			
RR/ MDR	**2173**	**0,5**	[19–35, 38, 39]
PRE- XDR	**104**	**0, 03**	[19–35, 38, 39]
XDR	**330**	**0,08**	[19–35, 38, 39]
Mono drug resistance (other than RIF)	**136**	**0,03**	[19–35, 38, 39]
Poli drug resistance)	**18**	**0,004**	[19–35, 38, 39]
STB/ controls	**2473**	**0,5**	[19–35, 38, 39]
DRTB type not indicated (no breakdown)	**408,542**	**98,7**	[36, 37, 40]
**Duration of study – Average duration of studies**	**32 months**	**–**	[19–40]
**Treatment outcomes**			
Favourable (Cured/ Treatment completed)	**89,410**	**21,60**	[29, 31, 33, 37]
Unfavourable (Died/ Defaulted/ LTFU/ Failed/ relapse)	**66,063**	**15, 96**	[29, 31, 33, 37]
Transferred out	**8**	**0,002**	[31]
Treatment outcome not evaluated in study	**258,295**	**62,42**	[30, 32, 34–36, 38–40]

## Results

### Search outcome

**Fig. 1:**
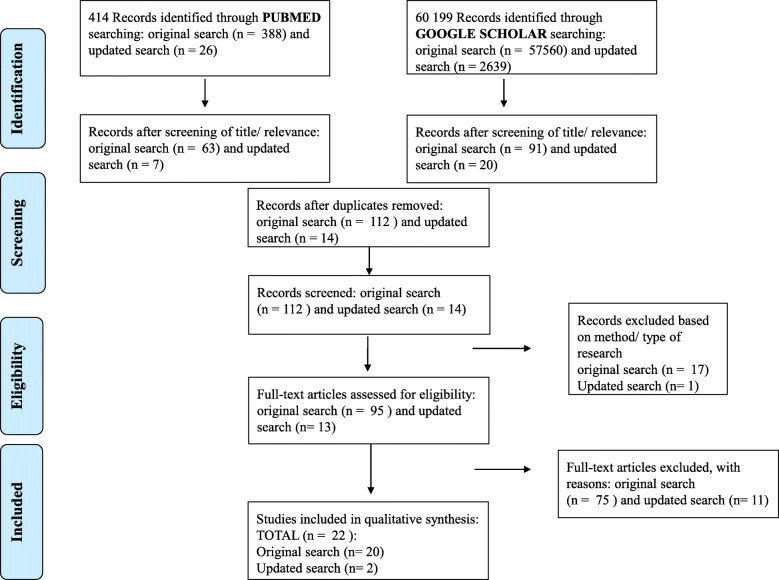
PRISMA flow diagram illustrating the search process which eligible articles was identified for data synthesis

The search process yielded 388 articles on PubMed and 57,560 on Google Scholar on the 7th January 2020 (Addendum 1). After screening of the titles of these records- 154 articles were selected. From the 154 articles retrieved, 91 were from Google Scholar and 63 from PubMed. Fourty-two of these were found to be duplications and removed. The 112 remaining articles were screened for appropriateness. Seventeen articles were removed based on qualitative study design. The 95 remaining articles (19 reviews and 76 quantitative studies), full papers were reviewed and further assessed for eligibility. For articles where the full text was not available, we used the Sci Hub website to retrieve the full text. Of the 95 articles whose full texts were screened, only 20 were found eligible and further assessed. Thus, a total number of 75 articles were further excluded as can be seen in Addendum 2. Out of the excluded articles, 59 were quantitative articles and involved 4 children< 5 years; 23 not Africa; 18 general/ Drug-Sensitive TB (DS-TB); 11 other factors- clinical; 1 isoniazid-resistant (INH) resistance; 2 XDR with unrelated content and 16 reviews excluded: 5 general- DS-TB; 4 other factors-clinical; 3 not applicable; 1 epidemiological study; 3 not systematic reviews. An updated database search was conducted on the 21st of September 2020 to identify the latest research on the topic. The search yielded 26 new articles in PubMed and 2639 in Google Scholar (Addendum 1). These articles were screened for eligibility as presented in Fig. 1. Only 2 of the articles were identified as eligible. Figure 1 below presents a schema for the search process and the outcome of the article selection process.

### Study setting of reviewed articles

**Fig. 2:**
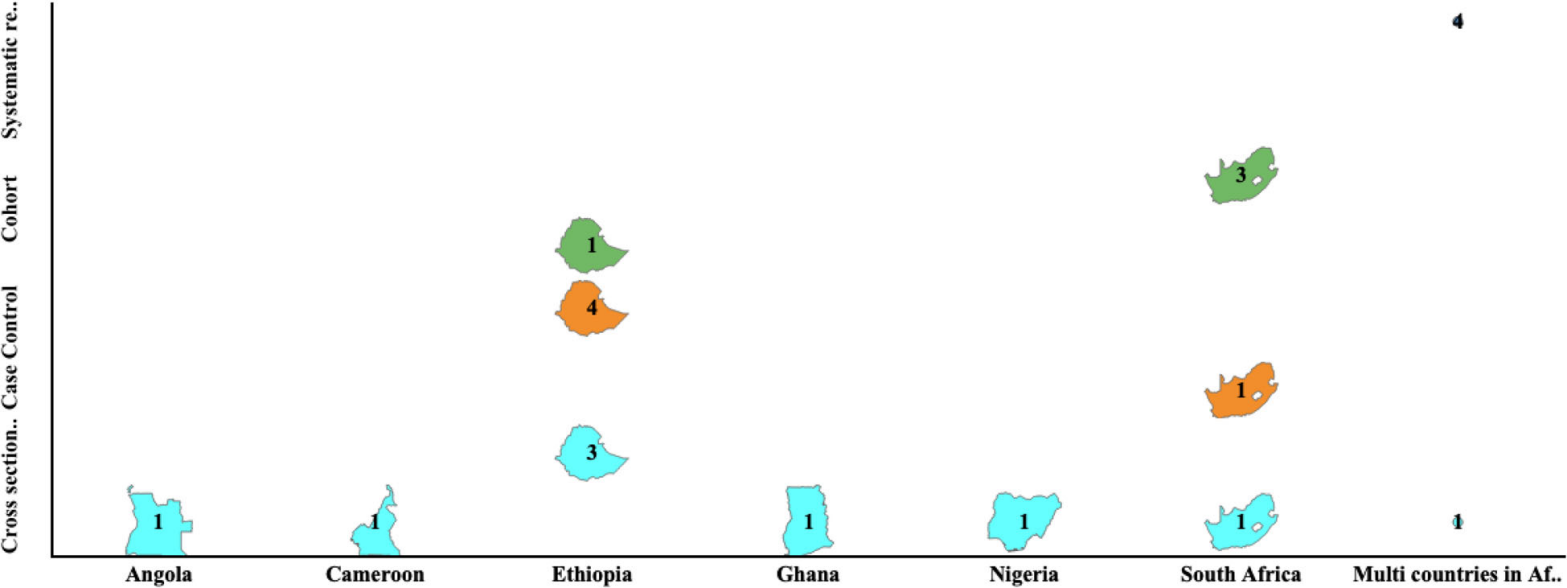
Study location and designs of all studies reviewed. Software used to design Fig. 2: Tableau 2020.4 [Internet]. 2020. Available from: https://www.tableau.com

Among the 20 eligible articles selected for review, eight studies (7) were conducted in Ethiopia, five (5) in South Africa and then respectively one (1) in each of the following countries: Cameroon, Nigeria, Ghana, and Angola. The three systematic reviews and one (1) cross- sectional study featured more than one nation all including Sub Sahara African countries. The 2 eligible articles in the updated search were done in Ethiopia (cross- sectional) and Sub Sahara African countries (systematic review). This is illustrated in Fig. 2.

### Summary of the studies reviewed

Table 1 presents the characteristics of the study population. Addendum 3 shows a summary of all 22 eligible articles, with specific focus on the main objectives and socio- economic factors that influence DR-TB. Of these selected studies, sixteen (17) were quantitative and three (3) were systematic reviews. Of the 17 quantitative articles, 5 were case controls, 4 cohort studies, 8 cross-sectional. These studies broadly focused on socio-economic risk factors, social issues, and financial issues. Eleven (11) of the articles reviewed focused on only social aspects, four (4) only on financial/ economic issues and five (5) articles covered both social and economic aspects. Of the selected articles, with the updated search one (1) was a cross- sectional study focusing on social aspects and a systematic review covering both social and economic aspects.

#### Emerged themes from studies reviewed

1. **Social issues influencing MDR-TB**

Thirteen studies identified in the initial search and both the studies in the updated search looked at the social issues influencing MDR-TB. This included apprehensions on stigma and prejudice, the impact of ethanol on the management of MDR-TB, low educational level, overcrowding, lack of treatment support and problems related to MDR-TB/HIV coinfection.

- **1.1 Substance use and abuse:** Five articles on treatment outcomes among the eligible studies found that alcohol consumption influenced MDR-TB. Alcohol consumption was found to be a predictor of MDR-TB poor treatment outcomes [21], while substance use was observed to cause poor adherence to treatment [40]. Hence, substance use and abuse were identified as risk factors for MDR-TB acquisition or development and conversely, alcohol consumption was associated with treatment default and failure rate among new DR-TB cases [28].
- **1.2 Poverty:** The highest rates of DS-TB and DR-TB were discovered in the disadvantaged settings of the community such as the rural areas, overcrowded households and congested areas [19, 23, 38]. Biru et al. (2020) found that residing in a one-roomed house is an independent factor related to DR-TB [36] . Low-income people and persons with little educational exposure had an increased risk of infection [35, 40]. Poverty may result in poor nutrition, which may be related, with alterations in the immune system, causing an increased vulnerability. Poverty, on the other hand, results in congestion, poor ventilation and unhygienic environments, increasing the risk of TB transmission [40].
- **1.3 Stigma and Discrimination:** Thomas et al. (2016) highlight stigma and discrimination as the drivers for the infection and transmission of MDR-TB [40]. Stigma and discrimination in MDR-TB burdened setting was shown to also affect a patient’s health-seeking behavior and access to healthcare negatively. Stigma and discrimination included rejection from family, friends or health workers, financial uncertainty and feelings of anxiety and depression []. It was found that MDR-TB patients would willingly isolate themselves for fear of infecting other members of the family. This can often resulted in isolation, cancellation of engagements, failed relationships and separation within the family [40].

2. **Economic Factors influencing MDR-TB**

MDR-TB has a vast financial effect on patients due to the complex nature and long duration and of treatment regimens. Socio-economic barriers affecting patient care included treatment distance, inaccessibility, transport costs and costs experienced during hospitalization [34]. Oga-Omenka et al. (2020) identified the inability to pay care-related costs as a barrier for diagnosis and treatment of DR-TB [37]. There was also associated job losses and production time loss during the initial intensive phase of treatment [24, 25]. Thomas et al. (2016) found that patients who had not returned to work after 1 year of being on medication were their family’s breadwinners which had to cease working for a substantial amount of time [40]. Decrease in income due to absenteeism from work and the treatment-related loss of income and extra costs was generally catastrophic [24].

### Quality of evidence

The Mixed Method Quality Appraisal Tool (MMAT) Version 2018 [41] was utilized to assess the quality of all the included quantitative studies (17) while, The Critical Appraisal Skills Programme (CASP) [42] tool was used to assess the systematic reviews. Two reviewers (LLC/ KEO) assessed the quality of the articles. The following categories were assessed: the appropriateness of the aim of the study, adequacy and methodology, study design, participant recruitment, data collection, data analysis, and findings presented. The quality score ranges from ≤50% as low quality, 51–75% an average quality, and 76–100% high quality. All the 17 included quantitative studies had high quality percentage of 76–100%. None of the included studies for quality assessment scored low quality. The case control study retrieved through the update search, also scored high quality The CASP has 3 domains which has 10 questions. Two of the reviews were found high quality and one average. The review generated from the updated search scored high quality with an overall of 70%. Quality assessment of evidence is attached as additional files (Addendum 4.1 and 4.2). The studies were considered to have minimal risk of bias.

## Discussion

This review specifically focused on the African region as this area has a high rate of DR- TB with limited resources to combat the disease burden. Thus, it is of utmost importance to identify these factors to stimulate the development of interventions to improve DR-TB treatment outcomes and decrease the incidence rate of DR- TB.

The social factors identified in the reviewed articles include apprehensions around stigma and prejudice, the impact of alcohol on the management of DR-TB, low educational level, overcrowding, and lack of treatment support. The social causes of TB are attributed to the mode of spread of TB, as well as the risk factors for acquiring the disease. This review highlights the social factors influencing DR-TB specifically emphasizing overcrowding as a major contributing factor. Eradicating extreme poverty and providing social protection could significantly reduce DR-TB. Patients with DR-TB would willingly isolate themselves for fear of infecting other members of the family, due to discrimination and stigma. This can often lead to separation, cancellation of engagements, failure of relationships and separation within the family [40]. In places like Africa where TB and HIV are still regarded as a “*killer disease”,* TB clients could suffer detrimental effects of being isolated, negatively affecting their treatment adherence status, thus, creating a risk for increased morbidity and mortality. Substance use is frequently observed among patients with poor adherence and has an impact on MDR-TB management. Substance abuse is also associated with high poverty and unemployment levels. In the population with an increased drug and alcohol utilization, it is empirical to study the impact of this increased substance abuse on MDR-TB and to identify intervention strategies to curb the problem.

The review also identified economic factors influencing DR-TB. Numerous factors were identified to cause financial constraints- job loss, loss of productive time, absenteeism, and regular reviews all due to the long duration of care and admission time. Breadwinners who are admitted cause significant emotional distress in families- these are also reasons for defaulting and absconding, to avoid admission- to be able to provide for the family. There are also treatment-related costs- getting to review date has associated transport cost. Serious effort needs to be made to address and develop strategies to decrease the financial burden.

There is, however, limited evidence to notify policymakers and TB control officers on how socioeconomic interferences should be used to improve MDR-TB control and reduce MDR-TB-related health disparities. There needs to be an immense scale-up of collaborative efforts toward the implementation of integrated model care, considering the health systems, socio-economic factors as well as medical factors.

### Limitations

Although a thorough literature search was conducted, only a small number of eligible articles were available. This limited number of articles could be because only two databases were used to retrieve information. The authors used these databases because they were robust open-access databases that required no institutional payments before they could be searched for eligible articles. Lastly, this scoping review focused on socio-economic factors that influence DR-TB in African regions. However, these factors could differ in other continents.

## Conclusion

In conclusion, this review has highlighted socio-economic factors that contribute to DR-TB. Furthermore, it is crucial to understand these socio-economic factors driving DR-TB in a context-specific setting, to come up with evidence-based intervention strategies that would curb the burden of DR-TB. To achieve the End TB strategy targets, it is imperative to harness both medical and socio-economic efforts to fight against DR-TB disease. DR-TB elimination would require a holistic, comprehensive approach, utilization of available strategies, and associated medical, socio and economic challenges. The management of DR-TB require concerted efforts that address the socio-economic factors as well. To strengthen the findings from this review and the body of evidence, larger-scale randomized control trials to test socio-economic interventions should be undertaken.

## Supplementary Information

**Additional file 1:.** Addendum 1: Search Strategy.

**Additional file 2:.** Addendum 2: Excluded studies with reasons.

**Additional file 3:.** Addendum 3: Summary of the main socio-economic and other factors associated with DR-TB in Africa.

**Additional file 4:.** Addendum 4.1: MMAT Quality Assessment Checklist.

**Additional file 5:.** Addendum 4.2: CASP Quality Assessment Checklists.

## Data Availability

All data generated and analyzed for the review are available upon request from the authors.

## Abbreviations

DS-TB: Drug-sensitive TB
DR-TB: Drug-resistance TB
LFT: Lost from treatment
MDR-TB: Multi-drug resistance TB
XDR-TB: Extensively drug resistance TB

## References

[1] Cramm JM, Finkenflügel HJM, Møller V, Nieboer AP (2010). TB treatment initiation and adherence in a south African community influenced more by perceptions than by knowledge of tuberculosis. BMC Public Health.

[2] Mcnally TW, De Wildt G, Meza G, Wiskin CMD (2019). Improving outcomes for multi-drug-resistant tuberculosis in the Peruvian Amazon–a qualitative study exploring the experiences and perceptions of patients and healthcare professionals. BMC Health Serv Res.

[3] World Health Organisation (2018). Global tuberculosis report 2018.

[4] Migliori GB, Richardson MDA, Sotgiu G, Lange C (2009). Multidrug-resistant and extensively drug-resistant tuberculosis in the west. Europe and United States: epidemiology, surveillance, and control. Clin Chest Med.

[5] Centre for Disease Prevention and Control (2017). Tuberculosis 2017.

[6] Khan R (2013). The social determinants of multidrug resistant tuberculosis in the United States between 2005 and 2009.

[7] World Health Organization (2019). Global tuberculosis report 2019.

[8] Ntoumi F, Kaleebu P, Macete E, Mfinanga S, Chakaya J, Yeboah-Manu D (2016). Taking forward the world TB day 2016 theme ‘unite to end tuberculosis’ for the WHO Africa region. Int J Infect Dis.

[9] World Health Organization (2015). Implementing the end TB strategy: the essentials.

[10] Manjelievskaia J, Erck D, Piracha S, Schrager L (2016). Drug-resistant TB: deadly, costly and in need of a vaccine. Trans R Soc Trop Med Hyg.

[11] Thiruvalluvan E, Thomas B, Suresh C, Sellappan S, Muniyandi M, Watson B (2017). The psychosocial challenges facing multi drug resistance tuberculosis patients: a qualitative study. J Tuberc Lung Dis HIV/AIDS.

[12] Rocha C, Montoya R, Zevallos K, Curatola A, Ynga W, Franco J (2011). The innovative socio-economic interventions against tuberculosis (ISIAT) project: an operational assessment. Int J Tuberc Lung Dis.

[13] Zhang C, Wang Y, Shi G, Han W, Zhao H, Zhang H (2015). Determinants of multidrug-resistant tuberculosis in Henan province in China: a case control study. BMC Public Health.

[14] Kaliakbarova G, Pak S, Zhaksylykova N, Raimova G, Temerbekova B, Hof S (2013). Psychosocial support improves treatment adherence among MDR-TB patients: experience from East Kazakhstan. Open Infect Dis J.

[15] Horter S, Stringer B, Reynolds L, Shoaib M, Kasozi S, Casas EC (2014). “Home is where the patient is”: a qualitative analysis of a patient-centred model of care for multi-drug resistant tuberculosis. BMC Health Serv Res.

[16] Lönnroth K, Jaramillo E, Williams BG, Dye C, Raviglione M (2009). Drivers of tuberculosis epidemics: the role of risk factors and social determinants. Soc Sci Med.

[17] Tricco AC, Lillie E, Zarin W, O'brien KK, Colquhoun H, Levac D (2018). PRISMA extension for scoping reviews (PRISMA-ScR): checklist and explanation. Ann Intern Med.

[18] Peters MDJ, Godfrey C, Mcinerney P, Baldini Soares C, Khalil H, Parker D. Chapter 11: scoping reviews. In: Aromataris E, Munn Z, editors. Joanna Briggs Institute Reviewer’s Manual: The Joanna Briggs Institute; 2017.

[19] Workicho A, Kassahun W, Alemseged F (2017). Risk factors for multidrug-resistant tuberculosis among tuberculosis patients: a case-control study. Infect Drug Resist.

[20] Gandhi NR, Andrews JR, Brust JCM, Montreuil R, Weissman D, Heo M (2012). Risk factors for mortality among MDR-and XDR-TB patients in a high HIV prevalence setting. Int J Tuberc Lung Dis.

[21] Mulisa G, Workneh T, Hordofa N, Suaudi M, Abebe G, Jarso G (2015). Multidrug-resistant mycobacterium tuberculosis and associated risk factors in Oromia region of Ethiopia. Int J Infect Dis.

[22] Mekonnen F, Tessema B, Moges F, Gelaw A, Eshetie S, Kumera G (2015). Multidrug resistant tuberculosis: prevalence and risk factors in districts of Metema and west Armachiho, Northwest Ethiopia. BMC Infect Dis.

[23] Roba AA, Dasa TT, Weldegebreal F, Asfaw A, Mitiku H, Teklemariam Z (2018). Tuberculosis patients are physically challenged and socially isolated: a mixed methods case-control study of health related quality of life in eastern Ethiopia. PLoS One.

[24] Van Den Hof S, Collins D, Hafidz F, Beyene D, Tursynbayeva A, Tiemersma E (2016). The socioeconomic impact of multidrug resistant tuberculosis on patients: results from Ethiopia, Indonesia and Kazakhstan. BMC Infect Dis.

[25] Ramma L, Cox H, Wilkinson L, Foster N, Cunnama L, Vassall A (2015). Patients' costs associated with seeking and accessing treatment for drug-resistant tuberculosis in South Africa. Int J Tuberc Lung Dis.

[26] Du Toit E, Squire SB, Dunbar R, Machekano R, Madan J, Beyers N (2015). Comparing multidrug-resistant tuberculosis patient costs under molecular diagnostic algorithms in South Africa. Int J Tuberc Lung Dis.

[27] Xavier PB, Peixoto B (2015). Emotional distress in Angolan patients with several types of tuberculosis. Afr Health Sci.

[28] Mulu W, Mekkonnen D, Yimer M, Admassu A, Abera B (2015). Risk factors for multidrug resistant tuberculosis patients in Amhara National Regional State. Afr Health Sci.

[29] Kendall EA, Theron D, Franke MF, Van Helden P, Victor TC, Murray MB (2013). Alcohol, hospital discharge, and socioeconomic risk factors for default from multidrug resistant tuberculosis treatment in rural South Africa: a retrospective cohort study. PLoS One.

[30] Meriki HD, Tufon KA, Atanga PN, Ane-Anyangwe IN, Anong DN, Cho-Ngwa F (2013). Drug resistance profiles of mycobacterium tuberculosis complex and factors associated with drug resistance in the northwest and southwest regions of Cameroon. PLoS One.

[31] Molie T, Teklemariam Z, Klinkenberg E, Dessie Y, Kumsa A, Mohammed H (2019). Intensive phase treatment outcome and associated factors among patients treated for multi drug resistant tuberculosis in Ethiopia: a retrospective cohort study. BMC Infect Dis.

[32] Oladimeji O, Ushie BA, Udoh EE, Oladimeji KE, Ige OM, Obasanya O (2016). Psychosocial wellbeing of patients with multidrug resistant tuberculosis voluntarily confined to long-term hospitalisation in Nigeria. BMJ Glob Health.

[33] Moyo S, Cox HS, Hughes J, Daniels J, Synman L, De Azevedo V, et al. Loss from treatment for drug resistant tuberculosis: risk factors and patient outcomes in a community-based program in Khayelitsha, South Africa. PloS One. 2015;10(3). 10.1371/journal.pone.0118919 PMC4364980 25785451

[34] Pedrazzoli D, Siroka A, Boccia D, Bonsu F, Nartey K, Houben R (2018). How affordable is TB care? Findings from a nationwide TB patient cost survey in Ghana. Tropical Med Int Health.

[35] Di Gennaro F, Pizzol D, Cebola B, Stubbs B, Monno L, Saracino A (2017). Social determinants of therapy failure and multi drug resistance among people with tuberculosis: a review. Tuberculosis..

[36] Biru D, Woldesemayat EM (2020). Determinants of drug-resistant tuberculosis in southern Ethiopia: a case–control study. Infect Drug Resist.

[37] Oga-Omenka C, Tseja-Akinrin A, Sen P, Mac-Seing M, Agbaje A, Menzies D (2020). Factors influencing diagnosis and treatment initiation for multidrug-resistant/rifampicin-resistant tuberculosis in six sub-Saharan African countries: a mixed-methods systematic review. BMJ Glob Health.

[38] Alene KA, Viney K, Mcbryde ES, Clements ACA (2017). Spatial patterns of multidrug resistant tuberculosis and relationships to socio-economic, demographic and household factors in Northwest Ethiopia. PLoS One.

[39] Lukoye D, Ssengooba W, Musisi K, Kasule GW, Cobelens FGJ, Joloba M (2015). Variation and risk factors of drug resistant tuberculosis in sub-Saharan Africa: a systematic review and meta-analysis. BMC Public Health.

[40] Thomas BE, Shanmugam P, Malaisamy M, Ovung S, Suresh C, Subbaraman R (2016). Psycho-socio-economic issues challenging multidrug resistant tuberculosis patients: a systematic review. PLoS One.

[41] Hong QN, Pluye P, Fàbregues S, Bartlett G, Boardman F, Cargo M, et al. Mixed methods appraisal tool (MMAT), version 2018. Registration of copyright, 2018; 1148552.

[42] Critical Appraisals Skills Programme. CASP Checklist: 10 questions to help you make sense of a Systematic Review. 2018. https://casp-uk.net/wp-content/uploads/2018/03/CASP-Systematic-Review-Checklist-2018_fillable-form.pdf.

