# Supplementary material for: Socio-economic drivers of drug-resistant tuberculosis in Africa: a scoping review
Source: BMC Public Health. 2021 Mar 11;21:488. doi: 10.1186/s12889-021-10267-0 (PMC7953648; doi:10.1186/s12889-021-10267-0)
Supplement: Supplementary file 1 — Additional file 1:. Addendum 1: Search Strategy. [file 12889_2021_10267_MOESM1_ESM.docx]

| **Database** | **Keywords** | **Search results from articles published between 1 Jan 2011 – 7 Jan 2020** | **Search results from articles published between 7 Jan 2020 – 21 Sep 2020** |
| --- | --- | --- | --- |
| PubMed  <https://www.nlm.nih.gov/bsd/pmresources.html> <https://pubmed.ncbi.nlm.nih.gov/> | drug resistant tuberculosis OR DRTB OR multidrug-resistant tuberculosis OR extremely/ extensively drug-resistant TB AND Africa AND socio-economic factors | 6 | 0 |
|  | drug resistant tuberculosis OR DRTB OR multidrug-resistant tuberculosis OR extremely/ extensively drug-resistant TB AND Africa AND social factors | 45 | 6 |
|  | drug resistant tuberculosis OR DRTB OR multidrug-resistant tuberculosis OR extremely/ extensively drug-resistant TB AND Africa AND economic factors | 121 | 5 |
|  | drug resistant tuberculosis OR DRTB OR multidrug-resistant tuberculosis OR extremely/ extensively drug-resistant TB AND Africa AND contributing factors | 26 | 4 |
|  | drug resistant tuberculosis OR DRTB OR multidrug-resistant tuberculosis OR extremely/ extensively drug-resistant TB AND Africa AND risk factors | 190 | 11 |
| Total |  | 388 | 26 |

| **Database** | **Keywords** | **Search results from articles published between 1 Jan 2011 – 7 Jan 2020** | **Search results from articles published between 7 Jan 2020 – 21 Sep 2020** |
| --- | --- | --- | --- |
| Google Scholar  <https://scholar.google.co.za/> | drug resistant tuberculosis OR DRTB OR multidrug-resistant tuberculosis OR extremely/ extensively drug-resistant TB AND Africa AND socio-economic factors | 3560 | 118 |
|  | drug resistant tuberculosis OR DRTB OR multidrug-resistant tuberculosis OR extremely/ extensively drug-resistant TB AND Africa AND social factors | 12100 | 486 |
|  | drug resistant tuberculosis OR DRTB OR multidrug-resistant tuberculosis OR extremely/ extensively drug-resistant TB AND Africa AND economic factors | 11100 | 467 |
|  | drug resistant tuberculosis OR DRTB OR multidrug-resistant tuberculosis OR extremely/ extensively drug-resistant TB AND Africa AND contributing factors | 14700 | 719 |
|  | drug resistant tuberculosis OR DRTB OR multidrug-resistant tuberculosis OR extremely/ extensively drug-resistant TB AND Africa AND risk factors | 16100 | 849 |
| Total |  | 57560 | 2639 |
