# Supplementary material for: Socio-economic drivers of drug-resistant tuberculosis in Africa: a scoping review
Source: BMC Public Health. 2021 Mar 11;21:488. doi: 10.1186/s12889-021-10267-0 (PMC7953648; doi:10.1186/s12889-021-10267-0)
Supplement: Supplementary file 2 — Additional file 2:. Addendum 2: Excluded studies with reasons. [file 12889_2021_10267_MOESM2_ESM.docx]

| **Database** | **Keywords** | **Search results from 1 Jan 2011 – 7 Jan 2020**  **Updated search 7 Jan 2020- 21 Sep 2020** | **Exclusion criteria** | **Number of articles retrieved after exclusion criteria applied** |
| --- | --- | --- | --- | --- |
| **PubMed**  <https://www.nlm.nih.gov/bsd/pmresources.html> <https://pubmed.ncbi.nlm.nih.gov/> | socio-economic factors, drug-resistance TB, multidrug-resistant tuberculosis, extremely/extensively drug-resistant TB, Africa, socio-economic factors social factors, economic factors, contributing factors, and risk factors | 388  Updated search= 26  (21 Sep 2020) | Exclude books & document  Screening title and abstract   - Updated search | 385  63  7 |
| **Google Scholar**  <https://scholar.google.co.za/> | socio-economic factors, drug-resistance TB, multidrug-resistant tuberculosis, extremely/extensively drug-resistant TB, Africa, socio-economic factors social factors, economic factors, contributing factors, and risk factors | 57560  Updated search= 2639  (21 Sep 2020) | Screening title and abstract   - Updated search | 91  20 |
| **PubMed and Google Scholar combined (after title/ abstract screened)**  Original and updated search | socio-economic factors, drug-resistance TB, multidrug-resistant tuberculosis, extremely/extensively drug-resistant TB, Africa, socio-economic factors social factors, economic factors, contributing factors, and risk factors | **181**  (154 + 27) | Records after duplicate removals | **126**  (112 + 14) |
|  |  | **126**  (112 + 14) | Studies excluded based on the method/ type of research (18 Qualitative studies) | **108**  (95 + 13) |
|  |  | **(95) Original search** | **59 Quantitative studies excluded:**   - 4 studies as it included children < 5 years of age - 23 studies were not done/ focused on Africa - 18 studies described general TB/ Drug-Sensitive TB (DS-TB) - 11 studies focussed on other factors non socio-economic factors/ clinical studies - 1 study focussed on Isoniazid-resistant (INH) resistance - 2 studies only on XDR with unrelated content   **16 Reviews excluded:**   - 5 studies described Drug-Sensitive TB (DS-TB) - 4 studies focussed on other factors non socioeconomic factors- clinical studies - 3 studies not applicable with unrelated content - 1 epidemiological study - 3 not systematic reviews | **(36)**  (91)  (68)  (50)  (39)  (38)  **(36)** |
|  |  | **(36) original search** |  | **(20)**  (31)  (27)  (24)  (23)  **(20)** |
|  |  | **(13) updated search** | **9 Quantitative articles excluded:**   - 1 study not available in English - 3 studies focussed on other factors non socioeconomic factors- clinical studies - 5 studies not relevant - 1 study describes general TB   **1 Review excluded:**   - 5 studies not relevant | **(3)**  (12)  (9)  **(4)**  **(3)** |
|  |  | **(3) updated search** |  | **(2)** |
|  |  | **TOTAL INCLUDED ARTICLES/ STUDIES** |  | **22** |
