# Supplementary material for: Socio-economic drivers of drug-resistant tuberculosis in Africa: a scoping review
Source: BMC Public Health. 2021 Mar 11;21:488. doi: 10.1186/s12889-021-10267-0 (PMC7953648; doi:10.1186/s12889-021-10267-0)
Supplement: Supplementary file 3 — Additional file 3:. Addendum 3: Summary of the main socio-economic and other factors associated with DR-TB in Africa. [file 12889_2021_10267_MOESM3_ESM.docx]

| First Author/ Reference | Year | Place | Method | Objective | Findings | Implications | Main associated socio-economic and other factors of MDR |
| --- | --- | --- | --- | --- | --- | --- | --- |
| Workicho et al. [[19](#_ENREF_19)] | 2017 | Ethiopia | Quantitative: Case-Control | Detecting the risk-factors for MDR-TB in patients who already have TB. | The following were strong forecasters for MDR-TB:   - Age - Previous treatment history - Living in a one-roomed house | MDR-TB strategies should focus on patients with:   - Young age - Infected with HIV - Previous treatment history - Crowded living environments. | Young age  Previous TB  HIV infected  Crowded living areas |
| Gandhi et al. [[20](#_ENREF_20)] | 2012 | South Africa | Quantitative: Case-Control | Identifying the risk factors related to death in MDR- and XDR-TB patients with HIV co-infection in South Africa. | Mortality in these patients was related to a larger level of immunosuppression and drug resistance. | Efforts to lessen the death rate should focus on:   - Strengthening TB management programs - Aggressive HIV testing and treatment initiation. | Immuno-compromised |
| Mulisa et al. [[21](#_ENREF_21)] | 2015 | Ethiopia | Quantitative: Case-Control | Determining risk factors for obtaining MDR-TB. | Predictors of MDR:   - Farmworker - TB contact - Alcohol - [HIV](https://www.sciencedirect.com/topics/immunology-and-microbiology/human-immunodeficiency-virus) infection - Previous TB / TB- treatment | In this region, the recognizing of high-risk patients and the diagnosis and treatment of MDR-TB should be a priority. | TB Contact  Farmworkers  Previous TB  Alcohol  HIV Infection |
| Mekonnen et al. [[22](#_ENREF_22)] | 2015 | Ethiopia | Quantitative: Cross-sectional | The objective is to determine the risk factors for MDR-TB and to estimate prevalence in this area. | Past anti-TB treatment usage was the only statistically noteworthy risk element for MDR-TB. | DOTS program and to optimizing diagnostic laboratory facilities should be improved. | Previous TB |
| Roba et al. [[23](#_ENREF_23)] | 2018 | Ethiopia | Quantitative: Case-control | This analysis aimed to evaluate the quality of life of MDR-TB patients in comparison (DS-TB) patients. | MDR-TB patients were more probable to have the following in comparison with DS-TB:   - Single - Current student - Low education level - Households with more than 5 persons   All of these were associated with poorer quality of life (p<0.05). | Suggested that healthcare facilities, media and all community participants be involved in the TB education of the community, homes and students. | Single  Student  Lower educational level  Crowded families >5 |
| Van den Hof et al. [[24](#_ENREF_24)] | 2016 | Ethiopia/ Kazakstan/ Indonesia | Quantitative: Cross-sectional | Measuring MDR-TB patients’ costs using a current TB patient cost measurement tool. | The economic burden of MDR-TB is distressing.  When the patient is the breadwinner the extra costs and loss of income impact worse on these families. | Relieving the cost of these patients should be a government priority. | Financial loss- unable to work  Job loss  No health insurance/ assistance  Loans |
| Ramma et al. [[25](#_ENREF_25)] | 2015 | South Africa | Quantitative: Cross-sectional | To approximation patient expenditures related to the diagnosis and treatment of RR-TB/MDR-TB in South Africa. | The average monthly patient costs accompanying the diagnosis and treatment of RR-TB and MDR-TB were greater during the intensive phase than the continuance phase (US$235 vs. US$188) and amongst in-patients than outpatients (US$269 vs. US$122). Most patients did not make use of social support for costs associated with the illness. | Suitable social support mechanisms should be offered to assist in managing these constraints. | Production time loss through hospitalization  Dietary enhancements requiring extra money  High out of pocket cost throughout the continuation phase  No social protection |
| Du Toit et al. [[26](#_ENREF_26)] | 2015 | South Africa | Quantitative: Cohort | An assessment of costs incurred by patients in LPA vs GeneXpert-centered diagnostic algorithms from symptom onset until treatment start for MDR-TB. | The average number of visits to initiation of MDR-TB treatment was reduced from 20 to 7 (*P*<0.001) and average costs fell from US$68.1 to US$38.3 (*P* = 0.004) in the Xpert group. The total of unemployed rose from 39% to 73% in the LPA group (*P*< 0.001) and from 53% to 89% in the Xpert group (*P*< 0.001). Average domestic income reduced by 16% in the LPA group and by 13% in the Xpert group. | The use of an Xpert-based process reduced the costs experienced by patients, but employment loss and income continue. Patients necessitate support to alleviate this impact. | Loss of employment and income |
| Xavier et al. [[27](#_ENREF_27)] | 2015 | Angola | Quantitative: Cross-sectional | The aim was to identify the levels of depression, anxiety, and emotional hardship in patients with TB. the secondary aim to establish the relationship between social-demographic and economic factors, clinical factors and anxiety, depression and emotional hardship. | 38.3% and 49.4% of the sample produced significant levels of anxiety and depression, res. 44.4% of patients had substantial degrees of emotional distress. Higher risks of anxiety reported in married patients. MDR-TB, diagnosis of extra-pulmonary TB. Female gender and cases of extra-pulmonary TB had a 1.5 times risk for emotional distress. | The study identified high rates of depression, anxiety, and emotional hardship amongst TB patients. Gender, Marital status, type and treatment of TB were related to greater emotional disorder levels. Mental health services should be an essential part of TB and MDR-TB care | Anxiety  Depression  Emotional distress  Marital and family problems/ separation |
| Mulu et al. [[28](#_ENREF_28)] | 2015 | Ethiopia | Quantitative: Case-Control | To determine the risk factors of MDR-TB patients in this setting. | Patients with TB treatment failure (AOR=13.5,CI=2.69-70), cavitations on chest x-ray (AOR=1.9,CI=1.1-3.38) and contact with MDR-TB patients (AOR=1.4,CI=0.19-0.39) were more probable to be MDR-TB patients. Other factors were:   - Low monthly salary (AOR=1.1, CI=0.34-0.47) - Alcohol intake (AOR=1.5, CI=0.2-0.98) - Young age (AOR=2.9, CI=1.07-7.68) | Strict devotion to directly observed therapy (DOTS), suitable management of TB patients and guidance on the nutrition are helpful to regulate the dissemination of MDR-TB. | Clinical factors: cavitations Previous TB failure  MDR-TB contact  Low monthly income  Alcohol usage  Young age |
| Kendall et al. [[29](#_ENREF_29)] | 2013 | South Africa | Quantitative: Cohort | Identify patient risk factors for defaulting from MDR-TB treatment and high-risk periods. | Defaulting was the main reason for patients not finishing treatment and those not cured. Other risks:   - Younger age - Financially- unstable patients - Alcohol and drug users | Outpatient care should be targeted to increase efforts to improve success rates. | Young age  Economically unstable  Alcohol and drug usage |
| Merikki et al. [[30](#_ENREF_30)] | 2013 | Cameroon | Quantitative: Cross-sectional | To studied anti-TB drug resistance reports, the influence of socioeconomic and as behavioural factors on the prevalence of TB and drug resistance. | Associated factors for TB:  Smoking  Alcohol consumption  Antiretroviral treatment for ≤ 12 months  Household TB contacts Only prior tuberculosis infection was linked with drug resistance. | Abstinence from smoking and alcohol should be incorporated in TB and HIV regulate programs by health authorities. | Smoking  Alcohol  ARVs < 12 months  Previous TB contact  Previous TB |
| Molie et al. [[31](#_ENREF_31)] | 2019 | Ethiopia | Quantitative: Cohort | The aim was to determine the outcome during the intensive phase and factors contributing to MDR-TB in Ethiopia. | Negative intensive phase treatment outcome was further present in:   1. Higher age [ARRR = 1.047, 95% CI (1.024, 1.072)] 2. History of hypokalemia [ARRR = 0.512, 95% CI (0.280, 0.939)].   Having an unknown intensive phase treatment outcome:   1. Managed under the ambulatory care [ARRR = 3.2, 95% CI (1.6, 6.2)], 2. Rural inhabitants [ARRR = 0.370, 95% CI (0.199, 0.66)] 3. No treatment supporter [ARRR = 0.022, 95% CI (0.002, 0.231)] | To better favourable treatment outcome stress should be given to:   1. Doing scheduled laboratory monitoring tests 2. Treatment adherence support 3. Recording and report 4. Quarterly cohort review 5. Elderly and rural patients | Older age  Rural residents |
| Oladimeji et al. [[32](#_ENREF_32)] | 2016 | Nigeria | Quantitative: Cross-sectional | Assessing the psychosocial wellbeing of MDR-TB patients in voluntary and remote long-term hospitalization in Nigeria. | Females had more psychosocial anxieties. The most common fears were:   1. Concern that people will get to know the patient infected bad type of TB (70%), 2. Dissatisfaction due to being separated from their marital partner (72%), 3. Anxieties about taking too many drugs (73%), 4. Irritation with not being able to continue social and economic activities (75%).   Those who were employed had more psychosocial concerns. Patients who had family support during hospitalization experienced a lower burden of psychosocial concerns. | Lengthy hospitalization stemmed in significant psychosocial burden for the MDR-TB patients. There is a need to consider alternative approaches with less psychosocial burden without compromising the quality of care. | Marital problems/ separation  Inability to continue socio-economic activities  Stigma |
| Alene et al. [[33](#_ENREF_33)] | 2017 | Ethiopia | Quantitative: Cross-sectional | Spatial analysis of MDR-TB, and its association with socio-economic, domestic factors and demographic in northwest Ethiopia. | Spatial crowding of MDR-TB was positively associated with:   1. Urbanization (RR: 1.02; 95%CI: 1.01, 1.04) 2. The percentage of men (RR: 1.58; 95% CI: 1.26, 1.99) | Cross-border projects including options for portable TB treatment units and follow up are vital for the successful control of MDR-TB in the region. | Spatial clustering  Periodic migration work |
| Moyo et al. [[34](#_ENREF_34)] | 2015 | South Africa | Quantitative: Cohort study involving a retrospective analysis of data | Investigating the loss from treatment (LFT), and post-treatment outcomes of DR-TB patients in this setting | LFT existed great, occurred throughout the therapy period and was mainly high in males and those aged 15-25 years. Overall, long-term survival was poor. | Further investigations are required to back the retention of DR-TB patients on treatment, even within community-based treatment programs. | Males  Young  Community-based care |
| Pedrazzoli et al. [[35](#_ENREF_35)] | 2018 | Ghana | Quantitative: Survey | Examining, by MDR class, the level and alignment of costs incurred by TB‐affected families; assessed the affordability of TB care; and describes surviving tactics used by TB‐affected homes. | Appalling costs affected 64.1% of patients. MDR more than DS patients. Payments for TB care led to a noteworthy increase of families in the study sample that live below the poverty threshold at the time of survey compared to pre‐TB diagnosis. | Innovative policies are urgently needed to certify TB care is affordable for TB patients. | High costs during treatment  Poverty |
| Di Gennaro et al. [[36](#_ENREF_36)] | 2017 | Multiple countries including African countries | Systematic Review | A review was directed at examining the affiliation of common social determinants on therapy failure and MDR in people with TB. | Therapy failure and MDR are linked to:   1. Low income 2. Low educational 3. Alcohol abuse | Further studies should focus on the effect of these social determinants on other evolving diseases and of other social determinants on TB prevention and treatment. should be implemented to prevent tuberculosis. | Low income  Low educational level  Alcohol abuse |
| Lukoye et al. [[37](#_ENREF_37)] | 2015 | Sub Saharan Africa | Systematic review | To evaluate the disparities in DR-TB and its determinants across SSA nations (new and formerly treated TB). | Pooled approximation of any DR-TB prevalence with the new cases was 12.6% (95% CI 10.6-15.0) while for MDR-TB this was 1.5% (95% CI 1.0-2.3). Among formerly treated patients, these were 27.2% (95% CI 21.4-33.8) and 10.3% (95% CI 5.8-17.4%), respectively. | Low prevalence of DR-TB in sub-Saharan Africa in comparison to the WHO estimates. MDR-TB in this region does not seem to be driven by the high HIV prevalence rates | Low DR-TB rates |
| Thomas et al. [[38](#_ENREF_38)] | 2016 | Rural Sub Saharan African (SSA) | Systematic review | Identify psychosocial factors influencing MDR-TB. | Issues reported causing psychosocial anxieties: Depression, stigma, discrimination, and financial limits due to MDR-TB. | a critical need exists for feasible, psychosocial and economic intervention studies that help to equip MDR-TB patients to cope with their illness, improve outcomes and quality of life of an MDR-TB patient | Depression  Stigma and discrimination  Psychological distress  Financial constraints |
| Biru et al. [41] | 2020 | Nigeria | Case control | The aim of this study was to determine the risk factors of DR-TB among TB patients in southern Ethiopia. | Living in a one-roomed house (adjusted odds ratio [AOR]: 6.8, 95% CI: 1.8–25.8), history of contact with DR-TB cases (AOR: 6.8, 95% CI: 1.8–25.3), treatment failure TB cases (AOR: 4.2, 95% CI: 1.1–15.5) and relapsed TB cases (AOR: 4.8, 95% CI: 1.3–18.1) were independent factors associated with DR-TB | Future studies should be done to include more DRTB patients to expand more on the current findings | Living in a one roomed house |
| Oga- omenka et al. [42] | 2020 | SSA | Systematic review | Aimed to identify barriers and facilitators in diagnosis and treatment of drug resistant tuberculosis (DR-TB) in SSA, | Principal patient-level barriers included loss to follow-up and death, as well as inability to pay care related costs. | Specific interventions targeting barriers may improve rates and timeliness of detection and treatment. | Geographic location  Life commitments  Ability to pay for transportation or services |
