# Supplementary material for: Socio-economic drivers of drug-resistant tuberculosis in Africa: a scoping review
Source: BMC Public Health. 2021 Mar 11;21:488. doi: 10.1186/s12889-021-10267-0 (PMC7953648; doi:10.1186/s12889-021-10267-0)
Supplement: Supplementary file 5 — Additional file 5:. Addendum 4.2: CASP Quality Assessment Checklists. [file 12889_2021_10267_MOESM5_ESM.docx]

**CASP Checklist: 10 questions to help you make sense of a Systematic Review**

| **Paper for appraisal and reference:** | **Di Gennaro, F., Pizzol, D., Cebola, B., Stubbs, B., Monno, L., Saracino, A., et al., Social determinants of therapy failure and multi drug resistance among people with tuberculosis: A review. Tuberculosis, 2017. 103: p. 44-51.** | **Lukoye, D., Ssengooba, W., Musisi, K., Kasule, G.W., Cobelens, F.G.J., Joloba, M., et al., Variation and risk factors of drug resistant tuberculosis in sub-Saharan Africa: a systematic review and meta-analysis. BMC public health, 2015. 15(1): p. 291.** | **Thomas, B.E., Shanmugam, P., Malaisamy, M., Ovung, S., Suresh, C., Subbaraman, R., et al., Psycho-socio-economic issues challenging multidrug resistant tuberculosis patients: a systematic review. PloS one, 2016. 11(1).** | **Oga-Omenka C, Tseja-Akinrin A, Sen P, et al. Factors influencing diagnosis and treatment initiation for multidrug-resistant/rifampicinresistant tuberculosis in six sub-Saharan African countries: a mixed-methods systematic review. BMJ Global Health 2020;5: e002280. doi:10.1136/ bmjgh-2019-00228** |
| --- | --- | --- | --- | --- |
| **1. Did the review address a clearly focused question?** | Yes | Yes | Yes | Yes |
| **2. Did the authors look for the right type of papers?** | Yes | Yes | Yes | Yes |
| **3. Do you think all the important, relevant studies were included?** | Yes | Yes | Not clear | No |
| **4. Did the review’s authors do enough to assess quality of the included studies?** | Yes | Not clear | Yes | Yes |
| **5. If the results of the review have been combined, was it reasonable to do so?** | Yes | Yes | Yes | No |
| **6.What are the overall results of the review?** | There was a significant unadjusted association between each of the social factors investigated and MDR. | The reported prevalence of DR-TB in SSA is low compared to WHO estimates. MDR-TB in this region does not seem to be driven by the high HIV prevalence rates. | Depression, stigma, discrimination, side effects of the drugs causing psychological distress, and the financial constraints due to MDR-TB were some of the common issues reported in the studies | Predominant patient-level barriers included loss to follow-up and death, as well as inability to pay care related costs |
| **7. How precise are the results?** | Low income (OR=167; 95%CI: 112-241, p=0006; I2 =80%; 14 studies; Supplementary Figure 6) and alcohol abuse (OR=188; 95%CI: 118-300, p=0008; I2 =70%; 7 studies were significantly associated with MDR, whilst low education was not. | Pooled estimate of any DR-TB prevalence among the new cases was 12.6% (95% CI 10.6-15.0) while for MDR-TB this was 1.5% (95% CI 1.0-2.3). Among previously treated patients, these were 27.2% (95% CI 21.4-33.8) and 10.3% (95% CI 5.8-17.4%), respectively. DR-TB (any and MDR-TB) did not vary significantly with respect to study characteristics | There is an urgent need for high-quality randomised controlled trials of psychosocial interventions for MDR-TB patients that focus on improving a wide variety outcome, including treatment outcomes (e.g., cure), mental health, and quality of life. (not clear) | There is a need for more studies focusing on contextual access dimensions and care cascades from more HBCs in SSA, as this review has highlighted a dominance of studies from South Africa |
| **8. Can the results be applied to the local population?** | Yes | Not clear | Not clear | Yes |
| **9. Were all important outcomes considered?** | yes | Yes | Not clear | Yes |
| **10. Are the benefits worth the harms and costs?** | Yes, if policies will address these factors and result in change | Yes | yes | Yes |
| **TOTAL out of 10** | 10 | 8 | 6 | 7 |
| **Percentage** | 100% | 80% | 60% | 70% |
